# Cause‐specific mortality after diagnosis of cancer among HIV‐positive patients: A collaborative analysis of cohort studies

**DOI:** 10.1002/ijc.32895

**Published:** 2020-03-12

**Authors:** Adam Trickey, Margaret T. May, M. John Gill, Sophie Grabar, Janne Vehreschild, Ferdinand W.N.M. Wit, Fabrice Bonnet, Matthias Cavassini, Sophie Abgrall, Juan Berenguer, Christoph Wyen, Peter Reiss, Katharina Grabmeier‐Pfistershammer, Jodie L. Guest, Leah Shepherd, Ramon Teira, Antonella d'Arminio Monforte, Julia del Amo, Amy Justice, Dominique Costagliola, Jonathan A.C. Sterne

**Affiliations:** ^1^ Population Health Sciences, University of Bristol Bristol United Kingdom; ^2^ Division of Infectious Diseases University of Calgary Calgary Alberta Canada; ^3^ Sorbonne Université, INSERM, Institut Pierre Louis d'Épidemiologie et de Santé Publique (IPLESP) Paris France; ^4^ Unité de Biostatistique et d'Épidémiologie Groupe Hospitalier Cochin Broca Hôtel‐Dieu, Assistance Publique Hôpitaux de Paris (AP‐HP), Université de Paris Paris France; ^5^ Department I for Internal Medicine University Hospital of Cologne Cologne Germany; ^6^ German Centre for Infection Research, Partner Site Bonn‐Cologne Cologne Germany; ^7^ Stichting HIV Monitoring Amsterdam The Netherlands; ^8^ Department of Global Health, Amsterdam University Medical Centers University of Amsterdam Amsterdam The Netherlands; ^9^ Amsterdam Institute for Global Health and Development Amsterdam The Netherlands; ^10^ University of Bordeaux, ISPED, INSERM U1219 Bordeaux France; ^11^ CHU de Bordeaux Bordeaux France; ^12^ Service of Infectious Diseases, Lausanne University Hospital Lausanne Switzerland; ^13^ University of Lausanne Lausanne Switzerland; ^14^ Department of Internal Medicine Antoine Béclère Hospital Clamart France; ^15^ University of Paris Saclay, Paris‐Sud University, UVSQ Le Kremlin‐Bicêtre France; ^16^ CESP INSERM U1018 Le Kremlin‐Bicêtre France; ^17^ Hospital General Universitario Gregorio Marañón, Instituto de Investigación Sanitaria Gregorio Marañón (IiSGM) Madrid Spain; ^18^ Medical University of Vienna Vienna Austria; ^19^ Rollins School of Public Health Atlanta GA; ^20^ Emory School of Medicine Atlanta GA; ^21^ Institute of Global Health, University College London London United Kingdom; ^22^ Unit of Infectious Diseases, Hospital Sierrallana Torrelavega Spain; ^23^ Clinic of Infectious Diseases and Tropical Medicine, San Paolo Hospital, University of Milan Milan Italy; ^24^ National Epidemiology Center Carlos III Health Institute Madrid Spain; ^25^ Yale University School of Medicine and Public Health New Haven CT; ^26^ VA Connecticut Healthcare System West Haven CT

**Keywords:** PLHIV, cohort, mortality, cancer, ADM, NADM

## Abstract

People living with HIV (PLHIV) are more likely than the general population to develop AIDS‐defining malignancies (ADMs) and several non‐ADMs (NADMs). Information is lacking on survival outcomes and cause‐specific mortality after cancer diagnosis among PLHIV. We investigated causes of death within 5 years of cancer diagnosis in PLHIV enrolled in European and North American HIV cohorts starting antiretroviral therapy (ART) 1996–2015, aged ≥16 years, and subsequently diagnosed with cancer. Cancers were grouped: ADMs, viral NADMs and nonviral NADMs. We calculated cause‐specific mortality rates (MR) after diagnosis of specific cancers and compared 5‐year survival with the UK and France general populations. Among 83,856 PLHIV there were 4,436 cancer diagnoses. Of 603 deaths after ADM diagnosis, 292 (48%) were due to an ADM. There were 467/847 (55%) and 74/189 (39%) deaths that were due to an NADM after nonviral and viral NADM diagnoses, respectively. MR were higher for diagnoses between 1996 and 2005 *versus* 2006–2015: ADMs 102 (95% CI 92–113) per 1,000 years *versus* 88 (78–100), viral NADMs 134 (106–169) *versus* 111 (93–133) and nonviral NADMs 264 (232–300) *versus* 226 (206–248). Estimated 5‐year survival for PLHIV diagnosed with liver (29% [19–39%]), lung (18% [13–23%]) and cervical (75% [63–84%]) cancer was similar to general populations. Survival after Hodgkin's lymphoma diagnosis was lower in PLHIV (75% [67–81%]). Among ART‐treated PLHIV diagnosed with cancer, MR and causes of death varied by cancer type, with mortality highest for liver and lung cancers. Deaths within 5 years of NADM diagnoses were more likely to be from cancer than AIDS.

## Abstract 2

**What's new?**

People with HIV live longer than they used to, thanks to advances in antiretroviral therapy. These improvements reduced the incidence of AIDS‐defining malignancies, such as Kaposi's sarcoma, but the increased life expectancy has led to more diagnoses of cancers not traditionally associated with HIV. Here, the authors studied cause‐specific mortality among people with HIV diagnosed with cancer. For those people, within 5 years after a cancer diagnosis, cause of death was more likely to be cancer than AIDS. Survival rates after diagnosis varied by cancer type, but were similar to rates among the general population.

**Abbreviations**
ADMs: AIDS‐defining malignancies
aMRR: adjusted mortality rate ratio
ART: antiretroviral therapy
ART‐CC: Antiretroviral Therapy Cohort Collaboration
CoDe: coding of death in HIV
COHERE: Collaboration of Observational HIV Epidemiology Research in Europe
HICDEP: HIV Cohorts Data Exchange Protocol
IQR: interquartile range
LTFU: lost to follow‐up
MRR: mortality rate ratio
NADMs: non‐AIDS‐defining malignancies
PLHIV: people living with HIV
SCC: squamous cell carcinoma

## Background

People living with HIV (PLHIV) are more likely to develop cancer than those not infected with HIV.^1^ This is the case not only for AIDS‐defining malignancies (ADMs), such as Kaposi's sarcoma and non‐Hodgkin's lymphoma^2^ but also for some non‐AIDS‐defining malignancies (NADMs) such as anal cancer.^3^, ^4^ The introduction of effective combination antiretroviral therapy (ART) in 1996 led to a large decrease in incidence of ADMs, because of control of HIV‐replication and improvement of immune status.^5^ With further improvements in HIV care, particularly starting ART earlier in the course of HIV disease, the incidence of ADMs has continued to decline.^6^ Improvements treatment of HIV have resulted in PLHIV living longer,^7^ increasing the risk of NADMs that are associated with age in the general population.^8^

The incidence of ADMs and NADMs among PLHIV has been well‐studied^2^, ^9^, ^10^, ^11^ but there is a lack of information on survival outcomes after specific cancer diagnoses. Few studies have analysed 5‐year survival after specific cancer diagnoses among PLHIV^12^, ^13^, ^14^, ^15^, ^16^, ^17^, ^18^, ^19^, ^20^ and they were limited to prognosis after diagnosis of ADMs,^12^, ^19^ or included untreated PLHIV^14^, ^15^ who have worse prognosis than those on ART.^5^ Little information is available on causes of death among PLHIV after diagnosis of cancer. Patterns of survival after cancer diagnosis are affected by several factors. Mortality rates vary considerably between different types of cancer and stages of cancer.^21^ In the general population, a death after a diagnosis of cancer may be more likely due to that cancer than in PLHIV because of the competing risks of death due to AIDS and other HIV‐related conditions. There are also differences in the demographics of those diagnosed with cancer in the general population compared to PLHIV, which may explain differences in patterns of death.^22^, ^23^

We studied cause‐specific mortality after diagnosis of specific cancers to answer the question: what do PLHIV diagnose with cancer die of? We also investigated changes over time in 5‐year survival after cancer diagnosis in PLHIV and compared this with survival in the general population of the United Kingdom and France.

## Materials and Methods

### Participating cohorts

Data were combined from 10 HIV cohorts from Europe and North America that participate in the Antiretroviral Therapy Cohort Collaboration (ART‐CC), which includes PLHIV aged ≥16 years who started ART after 1996.^24^ Ethics committees or institutional review boards approved the cohorts, which used standardised data collection methods, and followed‐up patients at least every 6 months. Included cohorts (listed in the supplement) had information on diagnoses of both ADMs (Kaposi's sarcoma, cervical cancer and non‐Hodgkin's lymphoma) and NADMs (all other cancers) and had information on cause of death available on ≥70% of deaths.

### Data on cancer and mortality outcomes

Information on cancers was captured through medical records at routine follow‐up and hospitalisation diagnostic codes. Cohorts validated cancer diagnoses, and provided dates of and reasons for hospitalisations, which were considered due to cancer if any of the reasons indicated cancer. Patients could be diagnosed with multiple types of cancer on the date of a hospitalisation. Available data on cancers was heterogeneous between cohorts. Six of the 10 cohorts included used medical records to gather information on cancer diagnoses, one cohort had linkage to a cancer registry, one provided treatment for both HIV and cancer in the same centre, one had cancer diagnoses reported to their database and verified by a clinician, and one had heterogeneous methods of collection across their sites (Supporting Information Table S1).

Cancer information in the form of ICD9 or ICD10 codes was translated to defined subcategories for cancers within HIV Cohorts Data Exchange Protocol (HICDEP) by AT, and then verified by a clinician (MJG). We classified oral cavity and pharynx squamous cell carcinoma (SCC), anal SCC, liver hepatocellular carcinoma, vagina SCC, vulva SCC, penis SCC and Hodgkin lymphoma as viral NADMs following Park *et al*.^25^ (Supporting Information Table S2).

Information on mortality was gathered through linkage with vital statistics agencies and hospitals or physician report, and the active follow‐up of participants. We used an adaptation of the Coding of Death in HIV (CoDe) project protocol (http://www.chip.dk/Tools-Standards/CoDe/About) to classify causes of death, as described previously.^26^ Deaths due to opportunistic infections secondary to complications of chemotherapy were classified as caused by cancer rather than infection or AIDS.

### Eligibility of patients and definition of follow‐up time

Eligible patients had a baseline CD4 cell count measured between 3 months before, and 2 weeks after, starting ART. Each cohort specified a date after which cancer data were deemed to be reliably collected: patients who initiated ART before then, or had cancer diagnoses before then, were excluded. Similarly, each cohort had a date before which cancer data were reliably collected: cancer diagnoses after this date, or patients starting ART following this, were excluded. Patients diagnosed with cancer before starting ART were excluded. Patients were followed up for a maximum of 5 years from the date of cancer diagnosis to the earliest of death, loss to follow‐up or cohort‐specific database administrative censoring. Patients with a gap of ≥1 year between date last known to be alive and administrative censoring were considered lost to follow‐up (LTFU) and were censored 6 months after their last recorded measurement. If a patient had multiple records of the same type of cancer, the second and subsequent records were assumed to be multiple instances of treatment or hospitalisations.

### Statistical analysis

We compared characteristics at start of ART and at cancer diagnosis of patients who were diagnosed with an ADM, viral NADM and nonviral NADM. Because of the substantial improvements in life expectancy of PLHIV since the introduction of ART, we stratified by period of cancer diagnosis (1996–2005 and 2006–2015).^7^ Individuals with diagnoses of multiple types of cancer could be in multiple groups. We tabulated the frequency of specific cancers by period of diagnosis.

We investigated causes of death after diagnosis of ADMs, viral NADMs, nonviral NADMs and after the most frequent cancers with ≥30 diagnoses during each calendar period (cervical cancer, head and neck cancers, Hodgkin's Lymphoma, liver cancer, Kaposi's Sarcoma, lung cancer and non‐Hodgkin's Lymphoma). Patients diagnosed with multiple cancers or cancer groups were included in the analysis for each cancer or cancer group, from the corresponding date of diagnosis. However, if both cancers were the same type (e.g. both NADMs), the patient was included only once in the analysis for that group, from the date of diagnosis of the first cancer. A patient could be included in an analysis for cancer having been diagnosed with another type of cancer before or during the follow‐up period: a sensitivity analysis included only patients diagnosed with one type of cancer. A sensitivity analysis also investigated excluding patients aged above 70 years of age at the time of cancer diagnosis.

For each cancer group, we used Cox survival regression models to estimate hazard ratios during the 5 years after cancer diagnosis, by period of cancer diagnosis (2006–2015 *vs*. 1996–2005) unadjusted and adjusted for CD4 count (0–99, 100–199, 200–349, 350–499, ≥500 cells/mm^3^), HIV‐1 RNA (0–499, 500–9,999, ≥10,000 copies/ml), and age (16–39, 40–49, 50–59, ≥60 years), all taken as the measurement closest to and before the date of cancer diagnosis, combined sex/transmission risk group, and stratified by cohort (ensuring that all comparisons are among patients in the same cohort).

For each cancer group and specific cancer, we used Poisson models to estimate mortality rates from all causes, AIDS, ADMs, NADMs, other causes (not cancer), and unclassifiable/unknown causes during the 5 years after cancer diagnosis, separately for 1996–2005 and 2006–2015. Other causes of death included all of the other coded deaths that were not due to AIDS or cancers, for example, hepatitis, cardiovascular, suicide, and so on. NADM deaths were not split as viral or nonviral NADMs as often detailed enough coding information was not available. We estimated both crude and standardised mortality rates; standardised to the ART‐CC population diagnosed with cancer, by age, sex and HIV transmission risk group. In the analysis of liver cancer, we added viral hepatitis as a specific cause of death because the CoDe system classifies deaths from cancer associated with hepatitis under hepatitis rather than NADM. Separately for each cancer group and by calendar period of diagnosis, we used a competing risks framework to generate 5‐year cause‐of‐death‐specific cumulative incidence functions, which we plotted in a stacked graph.

We calculated 5‐year survival after diagnosis of the seven most frequently occurring cancers between 2006 and 2015, and compared this to 5‐year cancer survival in the general population in the United Kingdom (2009–2013) (https://www.cancerresearchuk.org/health-professional/cancer-statistics/survival) and France (2005–2010),^27^, ^28^ standardised to the age and sex proportions of the ART‐CC population diagnosed with the cancer. Kaposi's sarcoma and non‐Hodgkin's lymphoma were excluded from this analysis as the burden among PLHIV drives the general population burden. For head and neck cancers combined, detailed information on 5‐year survival by age group was not available for the United Kingdom.

### Data availability

The data that support the findings of the study are property of the individual cohorts, which are listed on the ART‐CC website (http://www.bristol.ac.uk/art-cc/whoswho/). For data availability, the individual cohorts should each be contacted and data will be made available upon reasonable request, where applicable.

## Results

Records from 83,586 PLHIV providing 451,651 person‐years of follow‐up were analysed. We included 4,436 records of incident cancer for analysis, from 3,953 individual patients. Median follow‐up time was 3.3 years from first ADM diagnosis (interquartile range [IQR]: 0.7–5.0), while from first nonviral NADM diagnosis it was 1.2 years (IQR: 0.3–3.6), and from first viral NADM diagnosis 2.3 years (IQR: 0.6–5.0). Table 1 shows the numbers of malignancies of different types recorded in 1996–2005 and 2006–2015, as well as characteristics of patients according to type of malignancy. Both median age and median CD4 count at cancer diagnosis were lower for individuals diagnosed with an ADM than with an NADM, and for viral NADM compared to nonviral NADM. Both median age and median CD4 count at cancer diagnosis increased between 1996 and 2005 and 2006–2015, in each cancer group. The viral load when starting ART was similar across cancer groups and the two time periods, but the viral load at diagnosis of cancer was higher for patients diagnosed with ADMs than for those diagnosed with NADMs. Patients diagnosed with ADMs were more likely to have AIDS when starting ART than those diagnosed with NADMs. The most frequently occurring cancers were the ADMs Kaposi's sarcoma and non‐Hodgkin's lymphoma, while lung cancer and Hodgkin's lymphoma were the commonest NADMs (Table 2). Supporting Information Table S3 shows how characteristics, numbers of cancers and deaths varies by cohort.

**Table 1:** Numbers and characteristics of patients diagnosed with an AIDS‐defining malignancy (ADM), viral non‐AIDS defining malignancy (NADM) or a nonviral NADM, by calendar period of diagnosis

Characteristics at start of ART and at diagnosis of cancer	Cancer diagnosis 1996–2005			Cancer diagnosis 2006–2015		
	ADM (*n* = 1,091)	NADM viral (*n* = 186)	NADM nonviral (*n* = 424)	ADM (*n* = 1,071)	NADM viral (*n* = 441)	NADM nonviral (*n* = 996)
Age, years: median (IQR)						
ART start	38 (32, 47)	39 (34, 47)	43 (36, 53)	39 (32, 47)	41 (34, 48)	46 (38, 54)
Diagnosis of cancer	40 (34, 48)	43 (36, 50)	45 (39, 56)	42 (36, 50)	46 (40, 53)	51 (44, 60)
CD4 cells/mm^3^, median (IQR)						
ART start	125 (41, 264)	183 (68, 320)	160 (67, 301)	170 (60, 300)	201 (98, 322)	211 (82, 330)
Diagnosis of cancer	170 (64, 320)	239 (103, 408)	263 (119, 435)	238 (96, 416)	350 (185, 550)	414 (240, 631)
RNA copies/ml log median (IQR)						
ART start	5.1 (4.6, 5.6)	4.9 (4.2, 5.3)	5.0 (3.3, 5.5)	5.1 (4.6, 5.5)	4.9 (4.2, 5.4)	5.0 (4.4, 5.4)
Diagnosis of cancer	3.2 (2.3, 5.0)	2.5 (1.7, 4.1)	2.3 (1.7, 3.5)	2.4 (1.7, 4.7)	1.7 (1.6, 1.8)	1.7 (1.5, 1.7)
Sex and transmission risk group, *n* (%)						
MSM	549 (50%)	78 (42%)	140 (33%)	554 (52%)	225 (51%)	369 (37%)
Male PWID	68 (6%)	36 (19%)	60 (14%)	51 (5%)	54 (12%)	107 (11%)
Female PWID	34 (3%)	4 (2%)	17 (4%)	28 (3%)	9 (2%)	38 (3%)
Male heterosexual	184 (17%)	46 (25%)	95 (22%)	186 (17%)	82 (19%)	257 (26%)
Female heterosexual	144 (13%)	13 (7%)	67 (16%)	173 (16%)	40 (9%)	159 (16%)
Male other/unknown	88 (8%)	9 (5%)	41 (10%)	60 (6%)	24 (5%)	48 (5%)
Female other/unknown	24 (2%)	0 (0%)	4 (1%)	19 (2%)	7 (2%)	18 (2%)
AIDS at ART start						
No	526 (48%)	140 (75%)	281 (66%)	644 (60%)	329 (75%)	715 (72%)
Yes	565 (52%)	46 (25%)	143 (34%)	427 (40%)	112 (25%)	281 (28%)
Hepatitis C virus at ART start						
Negative	866 (79%)	126 (68%)	290 (68%)	859 (80%)	318 (72%)	739 (74%)
Positive	93 (9%)	41 (22%)	74 (17%)	91 (9%)	87 (20%)	141 (14%)
Missing/unknown	132 (12%)	19 (10%)	60 (14%)	121 (11%)	36 (8%)	116 (12%)
Ever smoked						
No	215 (20%)	28 (15%)	36 (8%)	340 (32%)	105 (24%)	218 (22%)
Yes	209 (19%)	58 (31%)	120 (28%)	335 (31%)	193 (44%)	469 (47%)
Missing/unknown	667 (61%)	100 (54%)	268 (63%)	396 (37%)	143 (32%)	309 (31%)

Abbreviations: ART, antiretroviral therapy; MSM, men who have sex with men; PWID, people who inject drugs.

**Table 2:** Cancers reported in the ART‐CC, diagnosed 1996–2005 and 2006–2015

Cancer type	*n*: 1996–2005	*n*: 2006–2015	*n*: Total
Kaposi's sarcoma	616	547	1,163
Non‐Hodgkin's lymphoma	449	464	913
Lung cancer	116	255	371
Hodgkin's lymphoma	109	196	305
Anal cancer	22	124	146
Cervical cancer	51	91	142
Head and neck cancers	42	96	138
Liver cancer	42	95	137
Leukaemia (all types)	28	61	89
Prostate cancer	16	62	78
Breast cancer	16	46	62
Bladder cancer	14	43	57
Connective tissue cancers	22	29	51
Malignant melanoma	10	40	50
Colon cancer	7	39	46
Brain cancer	17	19	36
Pancreatic cancer	5	25	30
Kidney/renal cancer	6	23	29
Stomach cancer	9	20	29
Rectal cancer	7	19	26
Uterus cancer	7	17	24
Testicular cancer	5	13	18
Penis cancer	4	12	16
Gynaecologic cancer	5	10	15
Bone cancer	6	7	13
Oesophageal cancer	2	10	12
Gall bladder cancer	2	4	6
Lip cancer	1	3	4

Not including metastasis, multiple myelomas, those of unspecified site, or unknown cancers.

### Prognosis after diagnoses of ADMs, viral NADMs and nonviral NADMs

Among 2,162 PLHIV who were diagnosed with an ADM 292/603 (48%) deaths were due to an ADM. Among 2,047 diagnosed with an NADM 467/847 (55%) deaths were due to an NADM. The proportions of deaths due to an NADM were 74/189 (39%) after a viral NADM diagnosis (*n* = 627) and 393/660 (60%) after a nonviral NADM diagnosis (*n* = 1,420).

Rates of mortality after ADM diagnosis were lower in the later period: the unadjusted mortality rate ratio (MRR) for 2006–2015 *versus* 1996–2005 was 0.73 (95% CI 0.61, 0.86) and the adjusted mortality rate ratio (aMRR) was 0.77 (0.65, 0.92). There was weaker evidence for such decrease for mortality after viral NADM diagnoses: MRR 0.75 (0.55, 1.02) and aMRR 0.76 (0.54, 1.07) and after nonviral NADM diagnoses: MRR 0.80 (0.68, 0.94) and aMRR 0.84 (0.70, 1.00)—seen in Table 3.

**Table 3:** All‐cause and cause‐specific mortality rates per 1,000 years (95% confidence intervals) during the 5‐years after diagnosis of (*i*) AIDS defining malignancy (ADM), (*ii*) viral non‐AIDS defining malignancy (NADM) and (*iii*) nonviral NADM, stratified by period of cancer diagnosis

	1996–2005			2006–2015			Unadjusted MRR^2^ (95% CI) for 2006–2015 *vs*. 1996–2005	Adjusted MRR^2^ (95% CI) for 2006–2015 *vs*. 1996–2005
Cause of death	Deaths	Crude rate	Standardised rate^1^	Deaths	Crude rate	Standardised rate^1^		
Diagnosis of ADM	*n* = 1,091 (889 Male, 202 Female)			*n* = 1,071 (850 Male, 221 Female)				
All	353	102 (92, 113)	77 (57, 103)	250	88 (78, 100)	73 (58, 93)	0.73 (0.61, 0.86)	0.77 (0.65, 0.92)
AIDS (not ADM)	83	24 (19, 30)	19 (16, 24)	41	15 (11, 20)	11 (8, 14)		
ADM	163	47 (40, 55)	34 (22, 52)	129	46 (38, 54)	37 (27, 52)		
NADM	15	4 (3, 7)	2 (0, 7)	8	3 (1, 6)	2 (1, 5)		
Other	36	10 (8, 14)	7 (5, 12)	32	11 (8, 16)	10 (8, 13)		
Unknown	56	16 (12, 21)	14 (11, 17)	40	14 (10, 19)	13 (8, 20)		
Diagnosis of viral NADM	*n* = 186 (169 Male, 17 Female)			*n* = 441 (385 Male, 56 Female)				
All	71	134 (106, 169)	98 (79, 122)	118	111 (93, 133)	86 (56, 134)	0.75 (0.55, 1.02)	0.76 (0.54, 1.07)
AIDS (not ADM)	7	13 (6, 28)	10 (7, 15)	6	6 (3, 13)	4 (1, 14)		
ADM	5	9 (4, 23)	12 (6, 22)	8	8 (4, 15)	8 (3, 20)		
NADM	35	66 (47, 92)	42 (21, 83)	39	37 (27, 50)	29 (11, 72)		
Other	19	36 (23, 56)	25 (15, 40)	41	39 (28, 52)	25 (17, 39)		
Unknown	5	9 (4, 23)	9 (3, 28)	24	23 (15, 34)	20 (12, 36)		
Diagnosis of nonviral NADM	*n* = 424 (336 Male, 88 Female)			*n* = 996 (781 Male, 215 Female)				
All	232	264 (232, 300)	222 (136, 363)	428	226 (206, 248)	213 (172, 263)	0.80 (0.68, 0.94)	0.84 (0.70, 1.00)
AIDS (not ADM)	20	23 (15, 35)	25 (19, 34)	13	7 (4, 12)	5 (3, 9)		
ADM	19	22 (14, 34)	21 (8, 54)	22	12 (8, 18)	13 (9, 19)		
NADM	133	151 (128, 180)	119 (62, 227)	260	137 (122, 155)	125 (95, 164)		
Other	40	46 (33, 62)	38 (25, 59)	40	21 (15, 29)	19 (14, 25)		
Unknown	20	23 (15, 35)	19 (14, 25)	91	48 (39, 59)	51 (36, 72)		

1 Standardised by sex/risk group and age to the ART‐CC population diagnosed with cancer.

2 Cox models stratified by cohort. Adjusted model contains sex/transmission risk category, and age, CD4 cells/mm^3^, and RNA copies/ml at cancer diagnosis.

Abbreviations: ADM, AIDS‐defining malignancy; CI, confidence interval; MRR, mortality rate ratio; NADM, non‐AIDS defining malignancy.

**Figure 1:**
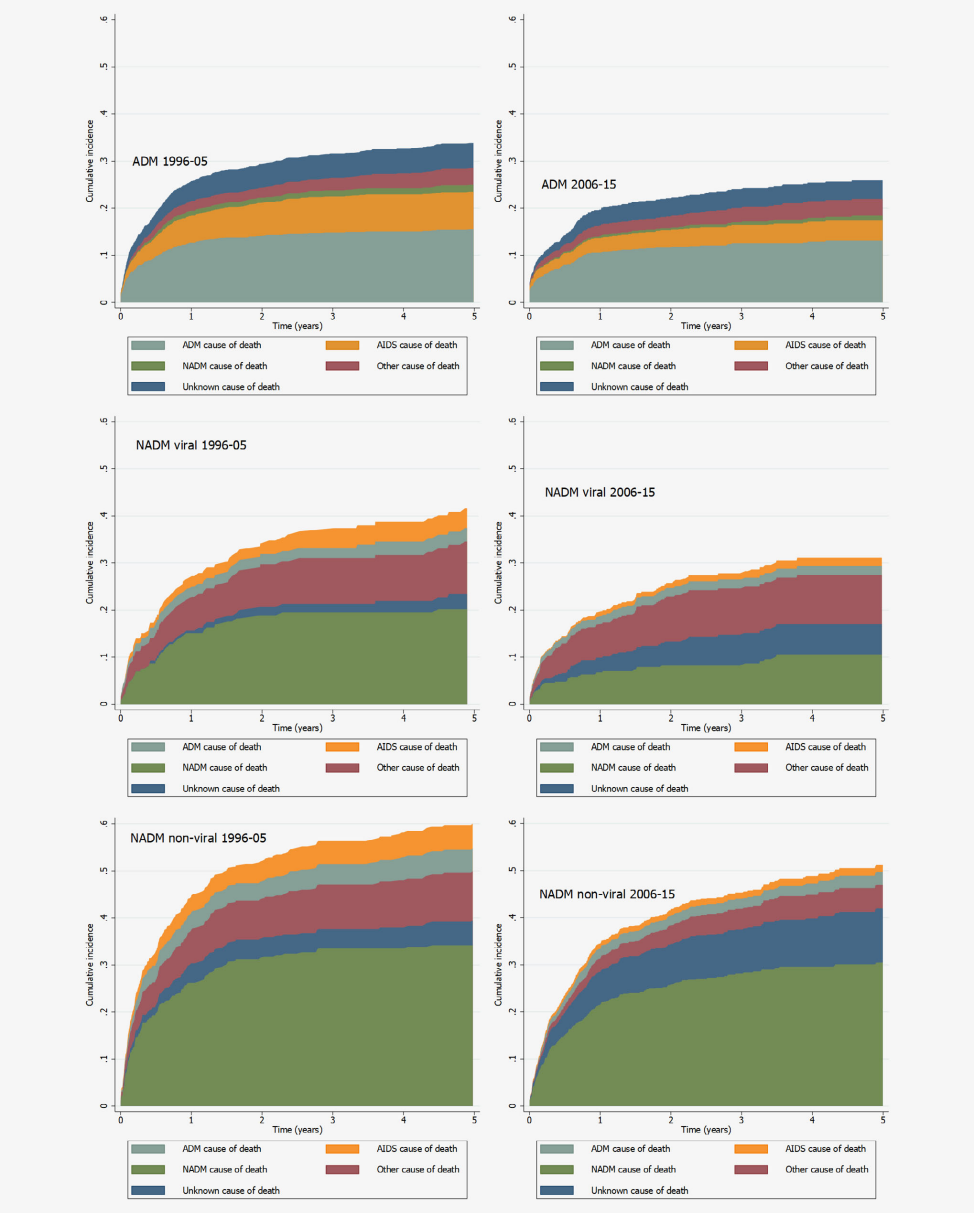
Cumulative cause‐specific mortality of patients from first diagnosis of ADM (upper), viral NADM (middle) and nonviral NADM (lower), stratified by period of cancer diagnosis (1996–2005 and 2006–2015). Abbreviations: ADM, AIDS‐defining malignancy; NADM, Non‐AIDS defining malignancy. Cumulative incidence functions were generating using a competing risks framework. [Color figure can be viewed at http://wileyonlinelibrary.com]

Table 3 shows crude and standardised rates of all‐cause and cause‐specific mortality during the 5 years after cancer diagnosis for the three cancer groups and by period of cancer diagnosis, with Supporting Information Table S4 giving more detailed information. Figure 1 shows patterns of 5‐year cumulative cause‐specific mortality according to type of cancer and calendar period. Among PLHIV diagnosed with an ADM, standardised rates and 5‐year cumulative incidence were greater for mortality due to an ADM than for other causes of death. AIDS was the second commonest cause of death in this group for both periods. Similarly, mortality due to NADMs was the leading cause of death among PLHIV diagnosed with nonviral NADMs. The pattern of causes of death for PLHIV diagnosed with viral NADMs was less clear: the standardised rate of deaths coded as ‘other’ was nearly as great as the standardised rate of deaths due to NADMs, particularly during 2006–2015. Many of these deaths had a cause classified as related to viral hepatitis. The proportion of PLHIV lost to follow up in the 5‐years after their cancer diagnosis was 16, 15 and 12% for those diagnosed with an ADM, viral NADM and nonviral NADM, respectively. Supporting Information Table S5 shows that there was little difference in mortality rates when patients diagnosed with more than one type of cancer were excluded from analyses, while Supporting Information Table S6 shows that there was also little difference excluding those aged over 70 years at the time of cancer diagnosis.

### Prognosis after diagnosis of specific cancers

Table 4 shows mortality rates for the seven most commonly diagnosed cancers: these were higher after diagnoses of NADMs than ADMs and were very high for lung, liver, non‐Hodgkin's lymphoma and head and neck cancers. For head and neck and lung cancer, the very high rates of NADM mortality suggest that cause of death was predominantly due to the diagnosed cancer. This was also the case for non‐Hodgkin's lymphoma, for which standardised rates of ADM mortality were high. A substantial proportion of deaths from liver cancer had been classified as due to viral hepatitis by our process for assigning CoDe cause of death classifications: these are shown as liver‐related in Table 4. Table 4 also contains unadjusted and adjusted MRRs for cancers diagnosed between 2006 and 2015 compared to 1996–2005. Evidence of reductions in MRRs between the two‐calendar year periods was seen for liver cancer: MRR 0.61 (95% CI 0.38, 1.00) and aMRR 0.41 (95% CI 0.22, 0.76), and non‐Hodgkin's lymphoma: MRR 0.62 (95% CI 0.51, 0.77) and aMRR 0.65 (95% CI 0.52, 0.81).

**Table 4:** All‐cause and cause‐specific mortality rates per 1,000 years (95% confidence intervals) during the 5‐years after diagnosis of cervical cancer, head and neck cancers, Hodgkin's Lymphoma, Kaposi’ sarcoma, liver cancer, lung cancer and non‐Hodgkin's Lymphoma

	1996–2005			2006–2015			Unadjusted MRR^2^ (95% CI) for 2006–2015 *vs*. 1996–2005	Adjusted MRR^2^ (95% CI) for 2006–2015 *vs*. 1996–2005
End point	Deaths	Crude rate	Standardised rate^1^	Deaths	Crude rate	Standardised rate^1^		
Cervical cancer	*n* = 51 (0 Male, 51 Female)			*n* = 91 (0 Male, 91 Female)				
All	8	39 (20, 79)	33 (16, 68)	16	60 (37, 98)	54 (35, 83)	1.44 (0.60, 3.46)	1.15 (0.42, 3.13)
AIDS (not ADM)	3	15 (5, 46)	21 (15, 30)	4	15 (6, 40)	9 (2, 34)		
ADM	3	15 (5, 46)	3 (0, 23)	7	26 (13, 55)	28 (20, 39)		
NADM	0	0 (0, 0)	0 (0, 0)	2	8 (2, 30)	9 (7, 13)		
Other	1	5 (1, 35)	5 (0, 47)	2	8 (2, 30)	3 (0, 33)		
Unknown	1	5 (1, 35)	5 (0, 47)	1	4 (1, 27)	4 (1, 25)		
Head/neck cancers	*n* = 42 (35 Male, 7 Female)			*n* = 96 (83 Male, 13 Female)				
All	18	182 (115, 289)	183 (71, 473)	36	175 (126, 242)	162 (108, 244)	0.76 (0.41, 1.41)	0.62 (0.31, 1.24)
AIDS (not ADM)	1	10 (1, 72)	20 (4, 115)	1	5 (1, 34)	2 (0, 14)		
ADM	0	0 (0, 0)	0 (0, 0)	1	5 (1, 34)	10 (1, 83)		
NADM	15	152 (91, 251)	136 (60, 310)	21	102 (67, 156)	90 (50, 160)		
Other	2	20 (5, 80)	27 (7, 104)	5	24 (10, 58)	15 (5, 44)		
Unknown	0	0 (0, 0)	0 (0, 0)	8	39 (19, 78)	46 (29, 72)		
Hodgkin's lymphoma	*n* = 109 (98 Male, 11 Female)			*n* = 196 (170 Male, 26 Female)				
All	28	74 (51, 107)	71 (57, 87)	35	64 (46, 89)	62 (37, 105)	0.68 (0.40, 1.15)	0.87 (0.48, 1.57)
AIDS (not ADM)	6	16 (7, 35)	13 (9, 20)	4	7 (3, 20)	7 (2, 25)		
ADM	4	11 (4, 28)	16 (9, 31)	7	13 (6, 27)	14 (5, 40)		
NADM	10	26 (14, 49)	17 (6, 45)	13	24 (14, 41)	21 (10, 47)		
Other	6	16 (7, 35)	23 (11, 48)	6	11 (5, 24)	10 (4, 26)		
Unknown	2	5 (1, 21)	2 (0, 9)	5	9 (4, 22)	9 (7, 13)		
Kaposi's sarcoma	*n* = 616 (542 Male, 74 Female)			*n* = 547 (489 Male, 58 Female)				
All	128	55 (46, 66)	46 (40, 54)	80	48 (39, 60)	43 (36, 51)	0.75 (0.56, 1.01)	0.91 (0.67, 1.22)
AIDS (not ADM)	46	20 (15, 27)	19 (14, 24)	19	12 (7, 18)	9 (6, 13)		
ADM	39	17 (12, 23)	14 (11, 20)	27	16 (11, 24)	16 (12, 21)		
NADM	4	2 (1, 5)	1 (0, 4)	2	1 (0, 5)	1 (0, 3)		
Other	19	8 (5, 13)	6 (4, 8)	19	12 (7, 18)	11 (7, 16)		
Unknown	20	9 (6, 13)	7 (4, 10)	13	8 (5, 14)	7 (4, 11)		
Liver cancer	*n* = 42 (39 Male, 3 Female)			*n* = 95 (76 Male, 19 Female)				
All	30	626 (434, 895)	324 (141, 744)	56	398 (306, 517)	401 (300, 536)	0.61 (0.38, 1.00)	0.41 (0.22, 0.76)
AIDS (not ADM)	1	21 (3, 148)	6 (1, 61)	1	7 (1, 50)	2 (0, 18)		
ADM	0	0 (0, 0)	0 (0, 0)	1	7 (1, 50)	3 (0, 24)		
NADM	17	355 (221, 571)	180 (63, 519)	11	78 (43, 141)	79 (27, 231)		
Liver‐related	8	167 (83, 334)	60 (14, 250)	31	220 (155, 313)	225 (171, 297)		
Other	3	63 (20, 194)	32 (1, 157)	2	14 (4, 57)	10 (2, 51)		
Unknown	1	21 (3, 148)	46 (14, 155)	10	71 (38, 132)	82 (54, 124)		
Lung cancer	N = 116 (101 Male, 15 Female)			N = 255 (217 Male, 38 Female)				
All	92	1,019 (831, 1,250)	791 (329, 1901)	184	752 (651, 869)	933 (756, 1,153)	0.81 (0.62, 1.06)	0.86 (0.65, 1.15)
AIDS (not ADM)	3	33 (11, 103)	17 (2, 123)	2	8 (2, 33)	19 (7, 54)		
ADM	2	22 (6, 89)	14 (2, 94)	1	4 (1, 29)	4 (1, 25)		
NADM	64	709 (555, 906)	557 (198, 157)	133	544 (459, 644)	669 (508, 882)		
Other	12	133 (76, 234)	106 (53, 214)	11	45 (25, 81)	46 (25, 83)		
Unknown	11	151 (110, 209)	96 (50, 183)	37	151 (110, 208)	195 (130, 292)		
Non‐Hodgkin's lymphoma	*n* = 449 (371 Male, 78 Female)			*n* = 464 (389 Male, 75 Female)				
All	234	237 (208, 269)	177 (121, 259)	168	173 (148, 201)	148 (107, 205)	0.62 (0.51, 0.77)	0.65 (0.52, 0.81)
AIDS (not ADM)	38	38 (28, 53)	23 (9, 55)	19	20 (12, 31)	15 (11, 21)		
ADM	130	132 (111, 156)	99 (69, 141)	104	107 (88, 129)	91 (62, 134)		
NADM	11	11 (6, 20)	5 (2, 16)	4	4 (2, 11)	3 (1, 7)		
Other	17	17 (11, 28)	13 (5, 30)	13	13 (8, 23)	10 (6, 18)		
Unknown	38	38 (28, 53)	37 (31, 44)	28	29 (20, 42)	28 (21, 38)		

1 Standardised by sex/risk group and age to the ART‐CC population diagnosed with cancer (by risk group and age to the female ART‐CC population for cervical cancer).

2 Cox models stratified by cohort. Adjusted model contains sex/transmission risk category, and age, CD4 cells/mm^3^ and RNA copies/ml at cancer diagnosis.

Abbreviations: ADM, AIDS‐defining malignancy; CI, confidence interval; NADM, Non‐AIDS defining malignancy; MRR, mortality rate ratio; MSM, Men who have sex with men.

**Figure 2:**
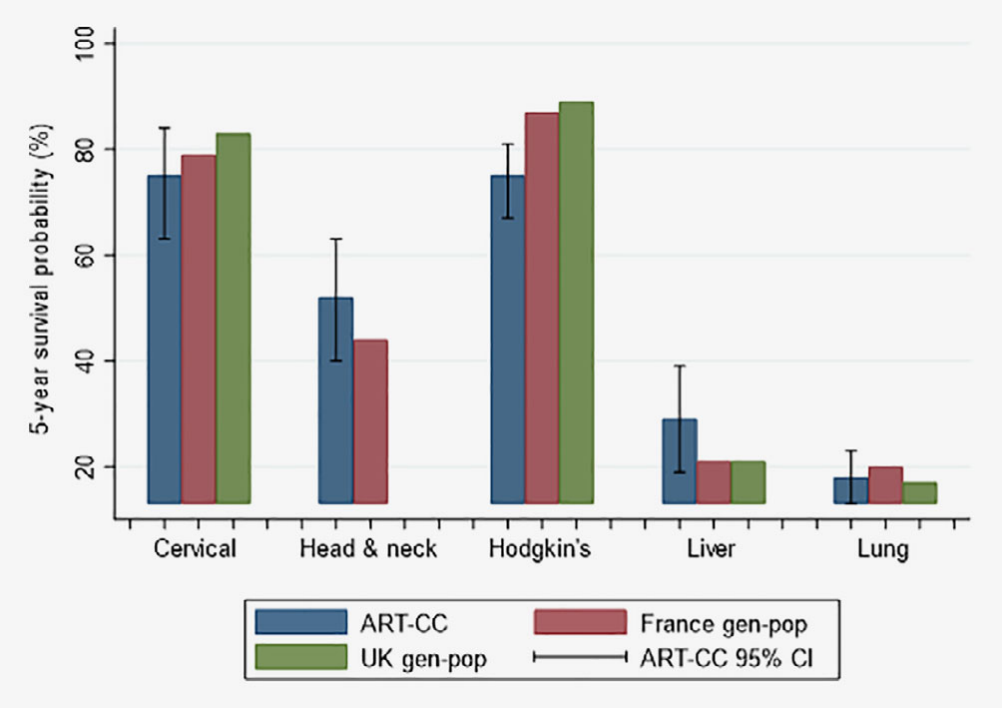
ART‐CC (all countries) and general population 5‐year survival percentages from the United Kingdom (UK) and France—standardised to the age and sex proportions of the ART‐CC population—for people diagnosed with selected cancers*. *CI: Confidence Interval; gen‐pop: general population. UK general population survival percentage not available for head and neck cancer. [Color figure can be viewed at http://wileyonlinelibrary.com]

Estimated 5‐year survival after diagnosis of cervical, head and neck, liver and lung cancer during 2006–2015 was similar in PLHIV to survival in people in the general population diagnosed with these cancers in the United Kingdom and France (Fig. 2). Five‐year survival for PLHIV diagnosed with Hodgkin's lymphoma was lower than in the general population diagnosed with that cancer.

## Discussion

Among PLHIV diagnosed with cancer, deaths due to an ADM were the leading cause of death after an ADM diagnosis while deaths due to an NADM were the leading cause of death after diagnosis of nonviral NADMs. Deaths after diagnosis with viral NADMs were most likely to be due to a NADM or due to other causes, many of which were classified as due to viral hepatitis. PLHIV diagnosed with nonviral NADMs had much higher mortality rates than those diagnosed with ADMs, or viral NADMs. Mortality rates after diagnosis of cancer were lower during 2006–2015 than 1996–2005: these declines were not explained by changes in CD4 count and viral load at cancer diagnosis. Possible explanations are earlier cancer stage at diagnosis due to improvements in screening,^29^ improvements in care among PLHIV,^7^, ^30^ greater awareness of drug–drug interactions,^31^ availability of more effective treatment for cancer^32^ or patients with higher CD4 counts being able to withstand more doses of chemotherapy.^33^

The most common cancers reported were the ADMs Kaposi's sarcoma, non‐Hodgkin's lymphoma, and cervical cancer and the NADMs lung cancer, Hodgkin's lymphoma, anal cancer, head and neck cancers and liver cancer. For lung cancer, head and neck cancer and non‐Hodgkin's lymphoma most deaths were likely to have been caused by the diagnosed cancer. Five‐year survival was low after diagnosis of liver cancer (as in the general population), but the underlying cause of death was often classified as viral hepatitis. Five‐year survival among PLHIV diagnosed with liver, lung and cervical cancers was similar to survival reported from general population cancer registries, but it was lower for PLHIV diagnosed with Hodgkin's lymphoma.

### Comparisons with other literature

To the best of our knowledge, our study is the first to analyse cause‐specific mortality among PLHIV diagnosed with a range of specific cancers, spanning multiple countries. A large registry‐linkage study in the USA found much higher mortality rates after diagnosis for comparable cancers and also found that a higher percentage of the mortality of PLHIV diagnosed with NADMs was due to AIDS.^34^ This difference could be due to earlier diagnosis of cancer in our cohorts compared to their registry‐linkage study, or because all of the patients in our analysis were on ART at the time of the cancer diagnosis, which was not necessarily the case in the USA study.^34^ A US study among elderly adults found higher cancer‐specific mortality for prostate and breast cancer for PLHIV compared to HIV‐negative people, however, we did not look at these individual cancers in our study.^35^ A small German study found that after just over a year, 87% of the deaths that occurred after diagnosis of lung cancer were due to lung cancer.^13^ This observation is concordant with our findings, although we had to infer that death due to NADM was due specifically to lung cancer. A general population study in the USA on diffuse large B‐cell lymphoma patients found that 24% of deaths were attributed to noncancer causes,^36^ a lower figure than in our study. However, mortality rates depend on the stage of cancer at diagnosis, age and demographics of the patients, which can vary between health systems, making comparisons difficult.

Several HIV cohort studies have estimated 5‐year survival after cancer diagnoses. Five‐year survival after diagnosis of non‐Hodgkin's lymphoma was lower in the Collaboration of Observational HIV Epidemiology Research in Europe (COHERE), a European study, than in ART‐CC (55% *vs*. 71%), but our study included more recent data.^16^ The Italian Cancer and AIDS registries linkage study found much lower 5‐year survival probabilities than ART‐CC after several specific cancers, for example, liver cancer 8% *vs*. 26%, but our study was during the early ART period and included PLHIV not on ART who had an AIDS diagnosis.^12^ Another Italian study which included PLHIV not on ART found similar 5‐year survival to ART‐CC after a diagnosis of Hodgkin's lymphoma.^14^ A separate Italian cohort study, which also included PLHIV not on ART estimated 4‐year survival concordant with our 5‐year survival for liver cancer, but much lower survival for Hodgkin's lymphoma (30% *vs*. 71%), lung cancer (6% *vs*. 16%), and cervical cancer (53% *vs*. 86%).^15^ A large French study which found improved survival between 2005 and 2009 compared to 1997–2000 for some haematological cancers and for ADMs, but not for solid cancers, reported higher 5‐year survival for Hodgkin's lymphoma than in ART‐CC (87% *vs*. 71%), and similar survival for lung and liver cancers.^17^ Compared to ART‐CC, a study in the USA found survival was lower for lung cancer (10% *vs*. 16%), and higher for Hodgkin's lymphoma (83% *vs*. 71%), possibly due to differences in the demographics between the two populations or the inclusion of PLHIV not on ART in the study from the USA.^18^

### Strengths and limitations

We analysed a large dataset of PLHIV receiving care in clinical cohorts in Western Europe and North America: our findings should be generalisable to PLHIV on ART in high‐income regions with recent cancer diagnoses. The size of our dataset enabled analyses of a wider range of specific cancers than previous studies. Most cohorts linked to death registries, but some PLHIV diagnosed with cancers with poor survival may have died after being lost to follow‐up, so we may have underestimated mortality rates.^37^ Information on cancer stage at diagnosis was not available: this may have resulted in some misclassification of precancerous lesions as cancer which could have biased survival estimates upwards. However, we focused our analyses on cancers that are more robustly validated and performed checks to remove precancers from the analysis.

We did not have conclusive data on cancer treatment such as chemotherapy and radiotherapy, which is a major prognostic factor.^32^ We also do not know if all people received the same standard of treatment across the different countries and regions.^21^ Survival comparisons with the general population may have been impacted by higher rates of smoking in PLHIV, but we were unable to adjust for this and other lifestyle factors.

Due to the lack of universal linkage to cancer registries, we could not examine cancer incidence as some diagnoses might not have been recorded by the cohorts. Cause‐specific mortality for PLHIV with recorded diagnosis of cancer should not be affected by this issue. We excluded those diagnosed with cancer who subsequently started ART due to uncertainty in timing of HIV infection and diagnosis. The cause of death information in ART‐CC was not specific enough to determine causes of death due to specific cancers, for example, some causes of death were only able to be coded as an NADM, rather than, for example, lung cancer. Therefore, our analyses are assuming that if, using the same example, a death was due to an NADM cancer after a diagnosis of lung cancer, then that death was most likely due to lung cancer. Additionally, for this analysis, we were unable to separate out deaths due to opportunistic infections caused by complications of chemotherapy as it was not available in HICDEP as a category of deaths. Classification of some deaths as AIDS‐related will have been based on a previous AIDS diagnosis, so some deaths may have been misclassified as due to infection rather than cancer, when the immediate cause was a complication of chemotherapy and the underlying cause, cancer, was not recorded. However, most cohorts recorded the underlying cause of death, or multiple causes of death, as well as the immediate cause.

### Implications

The improvement in survival of PLHIV diagnosed with cancer in 2006–2015 compared to 1996–2005 may reflect improvements in care among PLHIV, such as increased cancer screening resulting in earlier detection of cancers, which are easier to treat, or more effective cancer treatment.^32^ Another explanation could be improvements in ART resulting in better immunological status at cancer diagnosis, leading to more patients being able to tolerate chemotherapy.^33^

We reported estimates of 5‐year survival for several types of cancer. This information is important for clinicians and is easy to communicate to patients. The comparison with 5‐year survival in the general population quantifies the disparity that exists in some cancers and is a benchmark for future progress towards equalising survival rates. Although mortality rates decreased between 1996–2005 and 2006–2015, survival was worse for PLHIV diagnosed with Hodgkin's lymphoma than in the general population, possibly due to more aggressive forms of the cancer among PLHIV, interactions between ART and chemotherapy, or delayed use of new therapeutic options compared to the general population. More positively, for cervical, head and neck, liver and lung cancers there was no evidence of disparity in survival after cancer diagnosis between PLHIV and the general population. The publication and dissemination of such information may encourage PLHIV to be more proactive in being screened for cancer.

The population of PLHIV is ageing due to increased life expectancy attributable to more effective ART.^16^ As the cohort of PLHIV ages, they will be at increased risk of cancers not considered related to HIV that were previously rarely seen.^8^ As with the general population,^38^ many of these cancers are linked to lifestyle factors and comorbidities such as smoking and hepatitis C virus, both major sources of mortality among PLHIV.^39^, ^40^, ^41^ Emphasis should be placed on targeting such behaviours and treating co‐morbidities such as chronic viral hepatitis in order to prevent cancer.

## Conflict of interest

J.J.V. has personal fees from Merck/MSD, Gilead, Pfizer, Astellas Pharma, Basilea, Deutches Zentrum fur Infektionsforschung, Uniklink Freiburg/Kongress und Kommunikation, Akademie fur Infektionsmedizin, Universitat Manchester, Duetsche Fesellschaft fu Infektiologie, Arztekammer Nordrhein, Uniklinik Aachen, Back Bay Strategies, Deutsche Gesellschaft fur Innere Medizin and grants from Merck/MSD, Gilead, Pfizer, Astellas Pharma, Basilea, Deutsches Zentrum fur Infektionsforschung, Bundesministrium fur Bildung und Forschung. M.J.G. has received honoraria in the last 3 years from *ad hoc* membership of national HIV advisory boards, Merck, Gilead and ViiV. F.W.N.M.W. has received personal fees for HIV advisory board membership from ViiV. F.B. has received travel grants and honoraria from ViiV Healthcare, Gilead, BMS and MSD. His institution has received research grants from Gilead and ViiV Healthcare. D.C reports research grants from Janssen, MSD France, ViiV, personal fees from Janssen and MSD France for lectures, personal fees from ViiV for travel/accommodations/meeting expenses, personal fees from Gilead France for French HIV board, personal fees from Innavirvax and Merck Switzerland for consultancy, outside the submitted work. J.D.A. has received research grants and teaching fees from Gilead, ViiV Health Care, and MSD. M.C. reports that his institution received research grants from Gilead and ViiV. R.T. has received travel grants to attend medical meetings and/or speaker honoraria from Gilead, Janssen Cilag, MSD and ViiV Healthcare. P.R through his institution has received independent scientific grant support from Gilead Sciences, Janssen Pharmaceuticals Inc, Merck & Co and ViiV Healthcare, and has served on scientific advisory boards for Gilead Sciences, ViiV Healthcare, Merck & Co, Teva pharmaceutical industries, for which honoraria were all paid to his institution—none related to the content of this manuscript. All other members of the writing committee declare no competing interests.

## Supporting information


**Appendix S1**. Supporting Information.
