# Supplementary material for: Cause‐specific mortality after diagnosis of cancer among HIV‐positive patients: A collaborative analysis of cohort studies
Source: Int J Cancer. 2020 Mar 12;146(11):3134–46. doi: 10.1002/ijc.32895 (PMC7187452; doi:10.1002/ijc.32895)
Supplement: Supplementary file 1 — Appendix S1. Supporting Information. [file IJC-146-3134-s001.pdf]

Cause-specific mortality after diagnosis of cancer among HIV-positive patients: a collaborative analysis of cohort studies

Supplementary materials

Adam Trickey, *Margaret T* May, *M John* Gill, *Sophie* Grabar, *Janne* Vehreschild, *Ferdinand WNM* Wit, *Fabrice* Bonnet, *Matthias* Cavassini, *Sophie* Abgrall, *Juan* Berenguer, *Christoph* Wyen, *Peter* Reiss, *Katharina* Grabmeier-Pfistershammer, *Jodie L* Guest, *Leah* Shepherd, *Ramon* Teira, *Antonella* d'Arminio Monforte, *Julia* del Amo, *Amy* Justice, *Dominique* Costagliola, *Jonathan AC* Sterne

Table of contents

Page 2 - Cohorts included in analyses

Page 2 - Supplementary table S1: Cancer information sources for each cohort.

Page 3 - Supplementary table S2. ICD-O-3 topography and morphology code mappings for virus-NADC and poorly-specified cancers – reproduced from Park et al (2016).

Page 4 - Supplementary table S3: Characteristics by cohort.

Page 5 - Supplementary table S4: Detailed causes of death coding by cancer diagnosis group and calendar year period.

Page 6 - Supplementary table S5: All-cause and cause-specific mortality rates per 100000 years (95% confidence intervals) during the 5-years after diagnosis of: (i) AIDS defining malignancy (ADM), (ii) viral non-AIDS defining malignancy (NADM), and (iii) non-viral NADM, stratified by period of cancer diagnosis. Patients diagnosed with two or more cancers were excluded from the analysis.

Page 7 - Supplementary table S6: All-cause and cause-specific mortality rates per 100000 years (95% confidence intervals) during the 5-years after diagnosis of: (i) AIDS defining malignancy (ADM), (ii) viral non-AIDS defining malignancy (NADM), and (iii) non-viral NADM, stratified by period of cancer diagnosis. Patients aged over 70 years of age at cancer diagnosis were excluded from the analysis.

Cohorts included in analyses

Austrian HIV Cohort Study (AHIVCOS), Austria

ANRS CO3 Aquitaine Cohort, France

AIDS Therapy Evaluation project Netherlands (ATHENA)

Cohorte de la Red de Investigación en Sida (CoRIS), Spain

EuroSIDA

French Hospital Database on HIV (FHDH)

Italian Cohort of Antiretroviral-Naïve Patients (ICONA)

Köln/Bonn Cohort, Germany

South Alberta Cohort, Canada

VACH, Spain

See http://www.bristol.ac.uk/art-cc/whoswho/ for further information on these cohorts.

Supplementary table S1: Cancer information sources for each cohort.

Cohort	Source
AHIVCOS	Electronic medical record systems are installed in all hospitals providing specialised HIV care
Aquitaine	Cancer diagnoses are reported to the database and verified by a clinician
ATHENA	Cancer event information is collected directly from electronic patient files in the HIV treatment centers, with access also available to pathology reports and radiography results
Co-RIS	Cancer information is obtained through medical records
EuroSIDA	Heterogeneous across EuroSIDA's many sites
FHDH	Cancer information available based on review of histology retrieved from medical reports
ICONA	Cancer information is derived from data present in the clinical chart
Köln/Bonn	Almost all HIV patients are treated for their cancer at the same centre as treatment for HIV
South Alberta	Linkage to a cancer registry
VACH	Cancer information obtained from direct patient care and use of a comprehensive electronic medical record system

Supplementary table S2. ICD-O-3 topography and morphology code mappings for virus-NADC and poorly-specified cancers – reproduced from Park et al (2016).

Cancer group	Cancer type description	Related virus	ICD-O-3 topography code	ICD-O-3 morphology
Virus-NADC	Oral cavity and pharynx SCC	Human papillomavirus	C01.9 base of tongue<br>C02.4 lingual tonsil<br>C09.0-C09.9 tonsil<br>C10.0 vallecula<br>C10.2-C10.9 oropharynx, except anterior surface of epiglottis<br>C14.0 pharynx NOS<br>C14.2 Waldeyer ring	8050-8084, 8094 SCC<br>8121 Schneiderian carcinoma<br>8123 basaloid carcinoma<br>8010 carcinoma, NOS
	Anal SCC	Human papillomavirus	C21.0-C21.8 anus and anal canal<br>C20.9 rectum	8050-8084, 8094 SCC<br>8123 basaloid carcinoma<br>8124 cloacogenic carcinoma<br>8010 carcinoma, NOS[a]
	Liver hepatocellular carcinoma	Hepatitis C virus, hepatitis B virus	C22.0 liver	8170-8180 hepatocellular carcinoma
	Vagina SCC	Human papillomavirus	C52.9 vagina	8050-8084, 8094 SCC<br>8123 basaloid carcinoma<br>8010 carcinoma, NOS
	Vulva SCC	Human papillomavirus	C51.0-C51.9 vulva	8050-8084, 8094 SCC<br>8123 basaloid carcinoma<br>8010 carcinoma, NOS
	Penis SCC	Human papillomavirus	C60.0-C60.9 penis	8050-8084, 8094 SCC<br>8123 basaloid carcinoma<br>8010 carcinoma, NOS
	Hodgkin lymphoma	Epstein-Barr virus		9650-9667 Hodgkin lymphoma

ICD-O-3, International Classification of Diseases for Oncology, Third Edition; NOS, not otherwise specified; SCC, squamous cell carcinoma; virus-NADC, virus-related non-AIDS-defining cancers.

[a]Only for anal sites (C21.0-C21.8), not rectum (C20.9).

Supplementary table S3: Characteristics by cohort.

	N (% dead within 5 years)			Median (IQR)		% (% with missing data)		
**Cohort**	**ADMs**	**Viral NADMs**	**Non-viral NADMs**	**Age (years) at first cancer diagnosis**	**CD4 cells/mm³ at cancer diagnosis**	**AIDS at ART start**	**HCV at ART start**	**Ever smoked**
AHIVCOS	67 (31%)	33 (27%)	77 (39%)	46 (38, 55)	342 (195, 562)	36% (0%)	12% (9%)	46% (39%)
Aquitaine	81 (30%)	34 (38%)	86 (44%)	48 (38, 56)	329 (163, 549)	34% (0%)	14% (5%)	64% (26%)
ATHENA	411 (25%)	130 (38%)	341 (52%)	47 (39, 56)	333 (170, 540)	34% (0%)	7% (7%)	46% (19%)
CoRIS	58 (21%)	51 (12%)	67 (24%)	43 (35, 51)	346 (191, 553)	21% (0%)	15% (5%)	0% (100%)
EuroSIDA	54 (30%)	19 (32%)	43 (26%)	45 (36, 55)	307 (147, 540)	39% (0%)	16% (10%)	52% (17%)
FHDH	1076 (28%)	253 (29%)	451 (52%)	44 (37, 52)	212 (88, 391)	40% (0%)	13% (8%)	17% (60%)
ICONA	43 (37%)	26 (31%)	52 (38%)	44 (36, 52)	364 (184, 590)	18% (0%)	35% (6%)	64% (20%)
Köln/Bonn	32 (9%)	17 (24%)	23 (48%)	42 (36, 52)	255 (130, 438)	68% (0%)	1% (96%)	34% (63%)
South Alberta	71 (27%)	18 (28%)	46 (39%)	47 (37, 54)	295 (101, 496)	37% (0%)	10% (7%)	42% (25%)
VACH	269 (32%)	46 (30%)	234 (44%)	42 (35, 50)	262 (102, 475)	39% (0%)	14% (27%)	45% (39%)

IQR: Interquartile range; ADM: AIDS defining malignancy; NADM: Non-AIDS defining malignancy; ART: Antiretroviral therapy; HCV: Hepatitis C virus

Supplementary table S4: Detailed causes of death coding by cancer diagnosis group and calendar year period.

	**ADM**		**Viral NADM**		**Non-viral NADM**	
	**1996-2005**	**2006-2015**	**1996-2005**	**2006-2015**	**1996-2005**	**2006-2015**
AIDS	246 (70%)	170 (68%)	12 (17%)	14 (12%)	39 (17%)	35 (8%)
Cardiovascular	2 (1%)	3 (1%)	1 (1%)	1 (1%)	4 (2%)	3 (1%)
Liver	3 (1%)	3 (1%)	10 (14%)	31 (26%)	3 (1%)	6 (1%)
Non-AIDS infection	9 (3%)	11 (4%)	4 (6%)	2 (2%)	7 (3%)	8 (2%)
Non-AIDS, non-hep malignancy	12 (3%)	5 (2%)	34 (48%)	26 (22%)	121 (52%)	171 (40%)
Other	22 (6%)	14 (6%)	3 (4%)	17 (14%)	34 (15%)	101 (24%)
Respiratory	2 (1%)	2 (1%)	0 (0%)	2 (2%)	3 (1%)	10 (2%)
Substance abuse	0 (0%)	1 (0%)	2 (3%)	1 (1%)	1 (0%)	0 (0%)
Unknown/unclassifiable	56 (16%)	40 (16%)	5 (7%)	24 (20%)	20 (9%)	93 (22%)
Unnatural (Suicide/accident)	1 (0%)	1 (0%)	0 (0%)	0 (0%)	0 (0%)	1 (0%)
**Total**	**353**	**250**	**71**	**118**	**232**	**428**

Supplementary table S5: All-cause and cause-specific mortality rates per 100000 years (95% confidence intervals) during the 5-years after diagnosis of: (i) AIDS defining malignancy (ADM), (ii) viral non-AIDS defining malignancy (NADM), and (iii) non-viral NADM, stratified by period of cancer diagnosis. Patients diagnosed with two or more cancers were excluded from the analysis.

	1996-2005			2006-2015		
**Cause of death**	**Deaths**	**Crude rate**	**Standardised rate***	**Deaths**	**Crude rate**	**Standardised rate***
**Diagnosis of ADM**	**N=1066 (865 Male, 201 Female)**			**N=1024 (812 Male, 212 Female)**		
**All**	339	100 (90, 111)	76 (75, 76)	235	86 (76, 98)	72 (71, 72)
AIDS (not ADM)	81	24 (19, 30)	19 (19, 20)	38	14 (10, 19)	11 (10, 11)
ADM	155	46 (39, 53)	33 (33, 34)	119	44 (36, 52)	36 (35, 36)
NADM	12	4 (2, 6)	2 (2, 2)	8	3 (1, 6)	2 (2, 2)
Other	36	11 (8, 15)	8 (8, 8)	31	11 (8, 16)	10 (10, 11)
Unknown	55	16 (12, 21)	14 (13, 14)	39	14 (10, 20)	13 (13, 13)
**Diagnosis of viral NADM**	**N=175 (160 Male, 15 Female)**			**N=424 (368 Male, 56 Female)**		
**All**	63	122 (96, 156)	89 (87, 91)	113	109 (91, 131)	85 (84, 86)
AIDS (not ADM)	7	14 (6, 28)	11 (10, 11)	6	6 (3, 13)	4 (4, 4)
ADM	4	8 (3, 21)	8 (8, 9)	8	8 (4, 15)	8 (8, 9)
NADM	29	56 (39, 81)	34 (33, 35)	36	35 (25, 48)	28 (27, 29)
Other	18	35 (22, 55)	26 (25, 27)	39	38 (28, 52)	24 (23, 25)
Unknown	5	10 (4, 23)	10 (9, 11)	24	23 (16, 35)	21 (20, 21)
**Diagnosis of non-viral NADM**	**N=385 (305 Male, 80 Female)**			**N=913 (712 Male, 201 Female)**		
**All**	209	260 (227, 298)	235 (232, 238)	394	229 (208, 253)	219 (217, 220)
AIDS (not ADM)	17	21 (131 34)	24 (23, 25)	10	6 (3, 11)	5 (5, 5)
ADM	15	19 (11, 31)	22 (22, 23)	15	9 (5, 14)	11 (10, 11)
NADM	121	150 (126, 180)	124 (122, 126)	243	141 (125, 160)	129 (128, 130)
Other	37	46 (33, 63)	43 (42, 45)	35	20 (15, 28)	18 (17, 18)
Unknown	19	24 (15, 37)	21 (20, 22)	90	52 (43, 64)	56 (55, 57)

ADM: AIDS defining malignancy; NADM: Non-AIDS defining Malignancy; CI: Confidence Interval
*Standardised by sex/risk group and age to the ART-CC population diagnosed with cancer

Supplementary table S6: All-cause and cause-specific mortality rates per 100000 years (95% confidence intervals) during the 5-years after diagnosis of: (i) AIDS defining malignancy (ADM), (ii) viral non-AIDS defining malignancy (NADM), and (iii) non-viral NADM, stratified by period of cancer diagnosis. Patients aged over 70 years of age at cancer diagnosis were excluded from the analysis.

	1996-2005			2006-2015		
**Cause of death**	**Deaths**	**Crude rate**	**Standardised rate***	**Deaths**	**Crude rate**	**Standardised rate***
**Diagnosis of ADM**	**N=1076 (878 Male, 198 Female)**			**N=1052 (834 Male, 218 Female)**		
**All**	344	100 (90, 111)	76 (57, 102)	243	87 (77, 99)	72 (58, 91)
AIDS (not ADM)	82	24 (19, 30)	19 (15, 24)	40	14 (11, 20)	11 (8, 14)
ADM	159	46 (40, 54)	34 (22, 52)	127	45 (38, 54)	37 (26, 52)
NADM	14	4 (2, 7)	2 (1, 7)	8	3 (1, 6)	2 (1, 4)
Other	35	10 (7, 14)	8 (5, 12)	30	11 (8, 15)	10 (8, 12)
Unknown	54	16 (12, 21)	14 (11, 17)	38	14 (10, 19)	13 (8, 19)
**Diagnosis of viral NADM**	**N=178 (161 Male, 17 Female)**			**N=430 (376 Male, 54 Female)**		
**All**	65	128 (100, 163)	97 (79, 120)	112	107 (89, 129)	85 (55, 133)
AIDS (not ADM)	7	14 (7, 29)	10 (7, 15)	6	6 (3, 13)	4 (1, 14)
ADM	5	10 (4, 24)	12 (7, 22)	8	8 (4, 15)	8 (3, 20)
NADM	32	63 (44, 89)	42 (21, 82)	37	35 (26, 49)	29 (11, 73)
Other	17	33 (21, 54)	24 (15, 40)	38	36 (26, 50)	25 (16, 39)
Unknown	4	8 (3, 21)	9 (3, 28)	23	22 (15, 33)	19 (11, 34)
**Diagnosis of non-viral NADM**	**N=406 (320 Male, 86 Female)**			**N=920 (721 Male, 199 Female)**		
**All**	221	262 (229, 299)	221 (134, 363)	396	221 (201, 244)	212 (170, 263)
AIDS (not ADM)	19	23 (14, 35)	25 (19, 34)	12	7 (4, 12)	5 (3, 10)
ADM	18	21 (13, 34)	22 (8, 56)	22	12 (8, 19)	13 (9, 19)
NADM	128	152 (128, 180)	118 (61, 227)	243	136 (120, 154)	125 (95, 165)
Other	37	44 (32, 60)	38 (25, 59)	36	20 (15, 28)	18 (14, 24)
Unknown	19	23 (14, 35)	18 (14, 24)	81	45 (36, 56)	50 (34, 71)

ADM: AIDS defining malignancy; NADM: Non-AIDS defining Malignancy; CI: Confidence Interval
*Standardised by sex/risk group and age to the ART-CC population diagnosed with cancer
